# Comparison of respiratory pathogen yields from Nasopharyngeal/Oropharyngeal swabs and sputum specimens collected from hospitalized adults in rural Western Kenya

**DOI:** 10.1038/s41598-019-47713-4

**Published:** 2019-08-02

**Authors:** Bryan O. Nyawanda, Henry N. Njuguna, Clayton O. Onyango, Caroline Makokha, Shirley Lidechi, Barry Fields, Jonas M. Winchell, Jim S. Katieno, Jeremiah Nyaundi, Fredrick Ade, Gideon O. Emukule, Joshua A. Mott, Nancy Otieno, Marc-Alain Widdowson, Sandra S. Chaves

**Affiliations:** 1 Kenya Medical Research Institute - Center for Global Health Research, Kisumu, Kenya; 2 Centers for Disease Control and Prevention, Atlanta, GA USA; 3 Division of Global Health Protection, Centers for Disease Control and Prevention, Nairobi, Kenya; 4 Influenza Program, Centers for Disease Control and Prevention, Nairobi, Kenya

**Keywords:** Medical research, Statistics

## Abstract

Molecular diagnostic methods are becoming increasingly available for assessment of acute lower respiratory illnesses (ALRI). However, nasopharyngeal/oropharyngeal (NP/OP) swabs may not accurately reflect etiologic agents from the lower respiratory tract where sputum specimens are considered as a more representative sample. The pathogen yields from NP/OP against sputum specimens have not been extensively explored, especially in tropical countries. We compared pathogen yields from NP/OP swabs and sputum specimens from patients ≥18 years hospitalized with ALRI in rural Western Kenya. Specimens were tested for 30 pathogens using TaqMan Array Cards (TAC) and results compared using McNemar’s test. The agreement for pathogen detection between NP/OP and sputum specimens ranged between 85–100%. More viruses were detected from NP/OP specimens whereas *Klebsiella pneumoniae* and *Mycobacterium tuberculosis* were more common in sputum specimens. There was no clear advantage in using sputum over NP/OP specimens to detect pathogens of ALRI in adults using TAC in the context of this tropical setting.

## Introduction

Acute lower respiratory illnesses (ALRI) are associated with substantial morbidity and mortality in sub-Saharan Africa^1^. Understanding etiology of respiratory illnesses in resource-limited countries may support clinicians with patient management and provide a framework for decision makers when prioritizing public health interventions. Recently, with availability of multi-pathogen molecular diagnostic methods, and increasing recognition of viruses in cases of ALRI, the use of nasopharyngeal (NP) and oropharyngeal (OP) swab specimens to identify pathogens associated with ALRI has increased^2–4^. However, NP/OP swabs may not accurately reflect etiologic agents from the lower respiratory tract^5^. Sputum specimens, on the other hand, have been considered a more representative sample of the lower respiratory tract^5^, but have not been extensively explored, especially in tropical countries.

A multisite study in the United States compared pathogen yield between NP/OP and sputum specimens and argued for the use of high-quality sputum specimens for greater pathogen yield^6^. In contrast, another multisite study in low-middle income countries, observed that the pathogen yield from NP/OP and sputum specimens were similar^2^. Given the paucity of data and the difference in observations in these two recent studies, and that most data from tropical countries were derived from paediatric populations, we aimed to investigate adults living in a tropical, low-income setting. Here we describe the performance of NP/OP swabs against sputum specimens for detecting respiratory pathogens among adult patients hospitalized with ALRI using the TaqMan Array Card (TAC).

## Methods

### Study population and design

Since August 2009, the Kenya Medical Research Institute (KEMRI), in partnership with the U.S Centers for Disease Control and Prevention (CDC), has carried out surveillance for ALRI (defined as cough or difficulty breathing with an onset within the last 14 days from hospital admission date) among patients aged ≥ 18 years, at Siaya County Referral Hospital, in Western Kenya. The study population is culturally homogeneous and the area is known for high malaria, tuberculosis and HIV prevalence^7–9^. A structured questionnaire was programmed using Microsoft Visual Studio.net (Microsoft, Redmond, USA) and installed into netbooks (Mercer classmate) for collection of demographic and clinical examination data from patients by surveillance officers i.e. trained clinical officers and nurses. The data were stored in password protected Microsoft SQL databases (Microsoft, Redmond, USA). All patients were managed and discharged according to the Kenya Ministry of Health standards and guidelines.

### Specimen collection and processing

NP and OP specimens were collected from consenting patients meeting the ALRI case definition using flexible mini-tip flocked swabs, combined into a single vial containing viral transport media, and transported to the laboratory for testing as previously described^7^. Sputum specimens were collected through expectoration (quality sputum defined as <10 squamous epithelial cells per low-power field) in clean 3 ml tubes and transported at 2–8 °C to KEMRI/CDC laboratories in Kisumu where they were stored at −80 °C until testing. Nucleic acids were extracted from the sputum specimens, using a modified MagMax^TM^ (Applied Biosystems, Foster City, CA) viral RNA extraction procedure to include dithriothreital (DTT) treatment stage to break down sputum. Briefly, 100 μl of 500 mM DTT solution was added to 100 μl of sputum, vortexed and incubated at room temperature for 30 minutes. The MagMax^TM^ viral RNA extraction procedure was performed on 200 μl material following the manufacturer’s instructions. The extracted total nucleic acids from NP/OP and sputum were tested using TAC as described by Kodani *et al*. and Weinberg *et al*.^4,10^. Each target of the 30 pathogens was considered positive if it passed the internal positive control and had exponential fluorescence curves crossing the assigned threshold at a threshold cycle (C_T_) value of <35. For targets with two wells, the target was considered positive when either of the two wells was positive. Results with C_T_ > 30 for Legionella spp. were disregarded due to probe degradation of the specific chemistry used in preparation of this TAC.

### Data analysis

We described demographic characteristics and laboratory outcomes of patients using proportions, means, and medians. The paired specimen results were compared between NP/OP and sputum specimens using the McNemar’s test and further stratified by HIV status, malaria infection, clinical severity (≤ vs. >7 days hospitalized) and duration of illness (< vs. ≥5 days from onset to admission) which are provided as supplementary tables. Percent agreement between NP/OP and sputum specimens were calculated as the total number of times the NP/OP and sputum specimens agreed for each TAC target divided by the total number of patients tested. Tests with p-values <0.05 were considered statistically significant. The C_T_ values for each target were summarized using box-and-whiskers plots. Analysis was performed using SAS (version 9.2, Cary, NC).

### Ethical considerations

This study was reviewed and approved by the Kenya Medical Research Institute’s Scientific Ethics Review Unit (SSC no. 2558) and US Centers for Disease Control and Prevention’s Institutional Review Board (CDC IRB no. 6543). Written informed consents were obtained from all participants and all research was performed in accordance with the relevant guidelines/regulations.

### Disclaimer

The findings and conclusions in this article are those of the authors and do not necessarily represent the official position of the U.S. Centers for Disease Control and Prevention (CDC) or the Kenya Medical Research Institute (KEMRI).

## Results

From March 2014 through July 2015, we tested 294 pairs of NP/OP swabs and sputum specimens using TAC. On average, NP/OP swabs and sputum specimens were collected 29 (Standard Deviation [SD] = 14) and 38 (SD = 31) hours after admission, respectively.

Most patients (198/294; 67%) were between ages 18–49 years, and 81/294 (28%) were male. Fever was either self-reported or measured (≥38 °C) in 40% of the patients, and malaria was diagnosed in 19% of the patients. More than half the patients (179/294; 61%) had underlying chronic illness; 39% and 22% for HIV and other chronic conditions respectively. Of the 294 patients, 116 (39%) sought care in <5 days from respiratory illness onset, 99 (34%) sought care 5–10 days and 79 (27%) sought care after 10 days. Of all cases, 88 (30%) were hospitalized for >7 days and 24 (8%) died in hospital (Table 1). Of the 24 who died, 9 (38%) were HIV-positive, 3 (13%) had malaria and 3 (13%) had other chronic illnesses.

**Table 1:** Characteristics of patients hospitalized with acute respiratory illness at Siaya County Referral Hospital, March 2014–July 2015 (Total patient population N = 294).

Characteristics	n	%
**Age**^**a**^		
18 to <50 years	198	67
≥50 years	96	33
**Sex**		
Male	81	28
Female	213	72
**Signs and Symptoms at admission**		
Any fever	117	40
≥38 °C	27	9
Subjective	114	39
Headache	110	37
Cough	257	87
Difficulty in breathing	180	61
Sore throat	43	15
Rhinorrhoea	47	16
Chest pain	128	43
Night sweats	120	41
Diarrhoea	45	15
Lethargy	111	38
Jaundice	9	3
Tachypnea^b^	277	94
Hypoxia^c^	17	6
Wheeze	11	4
Stridor	27	9
**Severity of disease**		
Length of stay		
<3 days	78	26
3–7 days	128	43
>7 days	88	30
On oxygen	16	5
Death	24	8
**Co-morbidity**		
HIV	115	39
Chronic conditions (excluding HIV)^d^	64	22
Asthma	16	25
Diabetes	16	25
Cardiac disease	13	20
Malaria	57	19
**Health seeking behaviour**		
Hospitalised <5 days from onset	116	39
Hospitalised 5–10 days from onset	99	34
Hospitalised >10 days from onset	79	27

^a^Median (interquartile range) was 37 (28–56); ^**b**^Defined as respiratory rate >= 20; ^**c**^Defined as oxygen saturation <90% on room air; ^**d**^Presence of any of the following: chronic obstructive pulmonary disease, asthma, diabetes, chronic cardiac disease, chronic renal disease, chronic liver disease, chronic neurological impairment or any other immune compromising disease excluding HIV.

Of the 294 pairs of specimens tested, 248 bacterial and 136 viral pathogens were detected from 141 and 115 NP/OP specimens respectively whereas 213 bacterial and 47 viral pathogens were detected from 141 and 45 sputum specimens respectively. NP/OP specimens were more likely to test positive for any viral pathogen compared to sputum specimens, p < 0.01. There was no difference in bacterial pathogen detection in NP/OP vs. sputum specimens, and the pathogen specific agreements ranged between 85–100% (Table 2).

**Table 2:** Comparison of respiratory pathogen yields in nasopharyngeal/oropharyngeal (NP/OP) vs sputum specimens, March 2014–July 2015.

Pathogens	Positive		p-value	NP/OP & Sputum Positive	NP/OP & Sputum Negative	% Agreement Rate
	NP/OP	Sputum				
	n (%)	n (%)				
Any pathogen	200 (68)	170 (58)	<**0**.**01**	133	57	65
Any Bacterial pathogen	141 (48)	141 (48)	1	97	109	70
Any Viral pathogen	115 (39)	45 (15)	<**0**.**01**	38	172	71
**Bacterial pathogens**						
*Streptococcus pneumoniae*	76 (26)	61 (21)	**0**.**02**	49	206	87
*Haemophilus influenzae- All*	55 (19)	47 (16)	0.27	31	223	86
*Staphylococcus aureus*	41 (14)	28 (10)	0.07	12	237	85
Moraxella catarrhalis	35 (12)	22 (7)	**0**.**01**	17	254	92
*Pseudomonas aeroginosa*	18 (6)	19 (6)	1	3	260	89
Klebsiella pneumoniae	13 (4)	27 (9)	**0**.**01**	6	260	90
*Haemophilus influenzae B 1*	4 (1)	3 (1)	1	2	289	99
*Haemophilus influenzae B 2*	3 (1)	4 (1)	1	3	290	100
*Streptococcus pyogenes*	1 (0)	2 (1)	1	1	292	100
*Mycoplasma pneumoniae*	1 (0)	0		0	293	100
*Chlamydia pneumoniae*	1 (0)	0		0	293	100
*Bordetella pertussis*	0	0		0	294	100
*Legionella spp*.	0	0		0	294	100
**Viral pathogens**						
Rhinovirus	63 (21)	23 (8)	<**0**.**01**	21	229	85
Enterovirus	10 (3)	0		0	284	97
Influenza A	12 (4)	7 (2)	0.13	6	281	98
Influenza B	3 (1)	4 (1)	1	3	290	100
Resp. syncytial virus (RSV)	11 (4)	2 (1)	**0**.**01**	1	282	96
HCoV 2 (NL63)	9 (3)	1 (0)	**0**.**01**	1	285	97
HCoV 3 (OC43)	6 (2)	1 (0)	0.06	1	288	98
HCoV 4 (HKU1)	3 (1)	0		0	291	99
HCoV 1 (229E)	0	0		0	294	100
Para-influenza 3	5 (2)	2 (1)	0.25	2	289	99
Para-influenza 2	5 (2)	1 (0)	0.13	1	289	99
Para-influenza 4	2 (1)	0		0	292	99
Para-influenza 1	1 (0)	0		0	293	100
Human metapneumovirus	5 (2)	2 (1)	0.25	2	289	99
Adenovirus	1 (0)	4 (1)	0.38	0	289	98
**Mycobacteria**						
*Mycobacterium tuberclosis*	3 (1)	16 (5)	<**0**.**01**	2	277	95
**Fungi**						
*Pneumocystis jirovecii*	0	0		0	294	100

^*^Threshold cycle (C_T_) value of <35 considered positive, except for Legionella spp. where C_T_ > 30 for legionellae spp. were disregarded.

Among the bacterial pathogens detected, *Streptococcus pneumoniae* and *Moraxella catarrhalis* were more significantly present in NP/OP specimens compared to sputum specimens. However, *Klebsiella pneumoniae* and *Mycobacterium tuberculosis* were more significantly present in the sputum specimen compared to the NP/OP specimen. Among the viral pathogens, Respiratory Syncytial Virus (RSV), Rhinovirus, and Human Coronavirus 2 (NL63) were commonly detected in NP/OP than sputum specimens (Table 2). When stratified by HIV status, malaria infection, prolonged hospitalization and duration from onset to admission, there was not much difference in pathogen yield. Although, *Mycobacterium tuberculosis* (detected in sputum) was associated with HIV positive, malaria negative, and severe cases, and with patients admitted ≥5 days from illness onset. Also, patients with malaria were more likely to test positive for *Pseudomonas aeruginosa* from sputum compared to NP/OP specimens, p = 0.02 (Supplementary Tables S1–S4). *Streptococcus pneumoniae*, *Haemophilus influenzae* and *Moraxella catarrhalis* were the most co-detected bacterial pathogens and rhinovirus was the most co-detected viral pathogen (Supplementary Tables S5 and S6).

**Figure 1:**
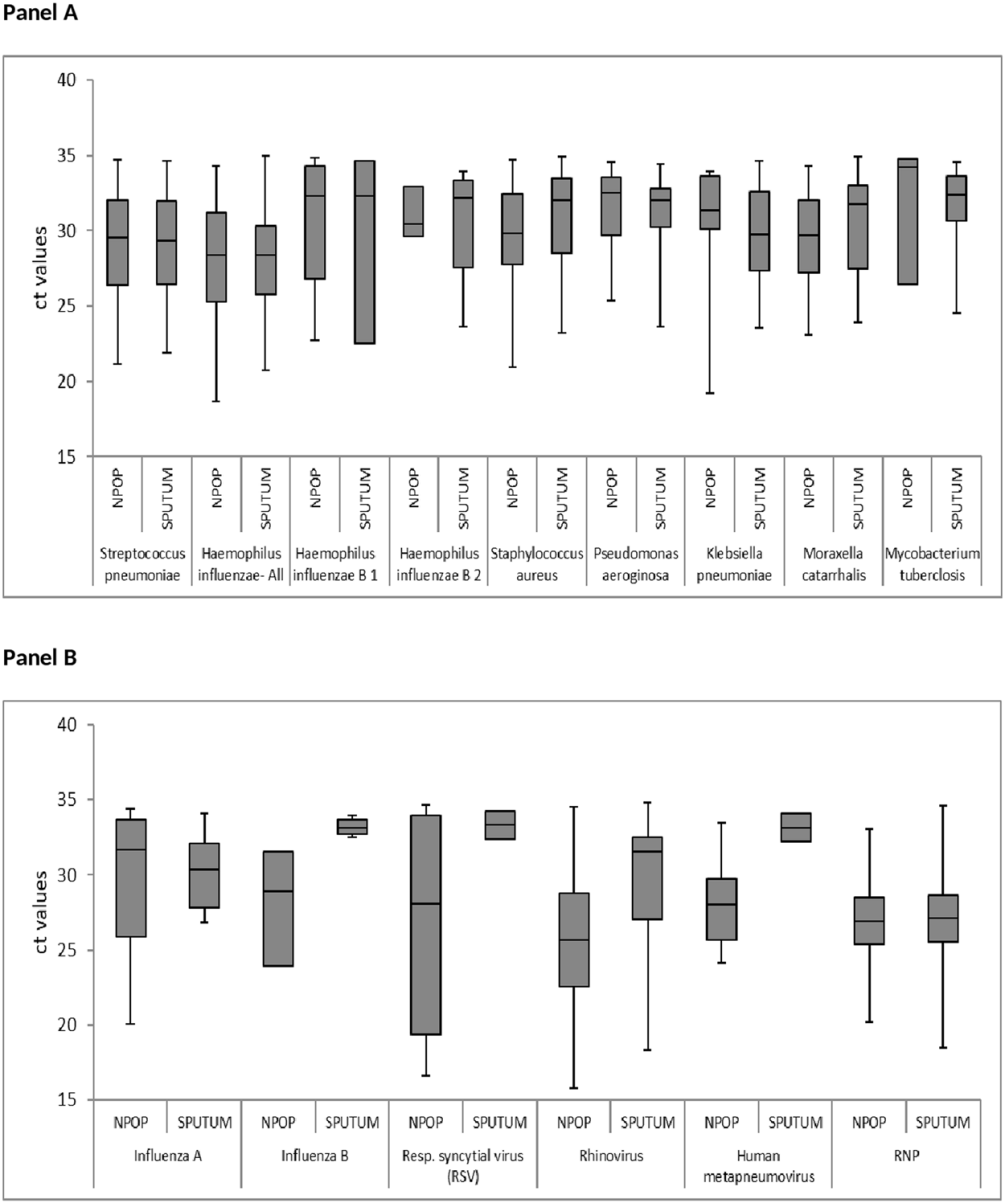
Box- Whiskers plot of threshold cycle (Ct) values for bacterial pathogens (panel A) and viral pathogens (panel B), detected by TaqMan Array Cards.

The median C_T_ values shown in box-and-whiskers plot were comparable for NP/OP and sputum specimens for all bacterial targets (Fig. 1). This was also observed for most viral pathogens, except that a higher concentration of viruses (corresponding to lower C_T_ values) was observed for influenza B, RSV and human metapneumovirus in the NP/OP specimens compared to sputum specimens. The median C_T_ values were similar in the NP/OP and sputum specimens for the RNP target.

## Discussion

We found good agreement (85–100%) in the detection of respiratory pathogens between NP/OP swabs and sputum specimens when using TAC. There was no overall benefit of using sputum over NP/OP swab specimens for etiologic assessment of respiratory illness, except that we found improved detection of *Mycobacterium tuberculosis* and *Klebsiella pneumoniae* from sputum specimens compared to NP/OP. When considering viral pathogens, rhinovirus, RSV, and coronavirus (HCoV NL63) were more often identified in NP/OP swabs. The use of molecular tests in NP/OP and sputum specimens warrants further investigation as a tool to identify etiology of ALRI in hospitalized adults.

Although previous pneumonia related studies have observed more bacteria yield from sputum^11,12^, arguing that this would be the ideal specimen for identification of lower respiratory tract infection etiology. We did not observe substantial differences in pathogen detection from NP/OP vs. sputum specimens. Mycobacterium tuberculosis, was mostly detected in sputum specimens, however, one case was detected in the NP/OP swab and not found in the sputum specimen. Though others suggested that this could happen in cases of extra-pulmonary tuberculosis^13^, we found that this patient was positive by GeneXpert (data not shown) which may suggest some shortfalls of testing for Mycobacterium tuberculosis in sputum using TAC.

Jeong *et al*. argued that as viruses play an important role in lower respiratory tract infections, sputum would be the best choice for etiologic diagnosis of virus-associated respiratory illness. The authors looked at adults who were hospitalized with severe acute respiratory illness, although the difference in yield from the two types of specimens was not impressive (15% increase in viral detection using sputum). Moreover, they only collected NP swabs which could have led to less representation of respiratory viruses with tropism to the oropharynx^14^. Wolff *et al*. also observed the detection of more viruses in sputum, where C_T_ values were earlier for almost all pathogens compared to that found in NP/OP swabs^6^, leading the authors to argue for the benefit of sputum use in diagnosis of lower respiratory tract infections. However this position was not supported in the recent work done by Thea *et al*.^2^, where they looked at children <5 years hospitalized with ALRI and pneumonia, or by our study done among adults. Perhaps in tropical countries, the likelihood of multi-pathogen detection may be higher than that seen in temperate countries, for children and adults alike, which may merit further investigation.

Both Wolff *et al*. and Thea *et al*. used high quality induced-sputum specimens in their studies but acknowledged that contamination would still be possible^2,6^. Furthermore, Thea *et al*. noted that the yield of pathogens from high quality induced sputum was similar to that of low quality induced sputum suggesting that specimen collected from the upper respiratory tract (i.e., NP/OP) could be used when investigating ALRI. The biggest challenge in these studies, including ours, is the interpretation of multi-pathogen detection, particularly when trying to attribute a specific pathogen as the cause of an episode of ALRI. Many bacterial pathogens detected from respiratory specimens could reflect carriage, being part of the normal microbiome, and not necessarily associated with the disease process. Similarly, viral pathogens detected in respiratory samples could indicate recent past infections. The early C_T_ values from the upper tract could indicate higher pathogen load and be associated with pneumonia^15^, which would suggest that NP/OP swabs could be used when investigating lower ALRI etiology if interpretation is combined with C_T_ values. However, establishing causality can be challenging even when considering organism density (here we used C_T_ values as proxy). The use of radiographic images, epidemiologic data, clinical course of illness, and the possibility to investigate lower respiratory tract specimens collected aseptically could be important tools to add to the investigation of ALRI/pneumonia.

A main limitation in our study is that direct comparison of our findings with those from the literature is complicated due to varying case definitions, severity of cases investigated, age of patients, study settings (temperate vs. tropical, less vs. more resourced countries), and laboratory tests used. Secondly, we did not use induced sputum collection as we worked with a population of adult patients, most of whom were accustomed to providing sputum specimen for tuberculosis testing. Moreover, as used by Thea *et al*.^2^, we considered <10 squamous epithelial cells per low-power field to determine specimen quality and observed that RNP C_T_ values were similar for NP/OP and sputum specimens, giving us confidence that results from sputum specimens were credible.

In conclusion, our findings suggest that there is no clear advantage in using sputum specimens over NP/OP swabs to investigate the etiology of ALRI using molecular diagnostics. Interpretation of multi-pathogen molecular laboratory tests, however, needs further investigation, especially in tropical settings where co-detections seem very common.

## Supplementary information

Supplementary Tables

## Data Availability

All the data are included in this manuscript through tables, figures and supplementary tables but should additional data be required, requests can be made to the director KEMRI through the corresponding author.
