# Supplementary material for: Comparison of respiratory pathogen yields from Nasopharyngeal/Oropharyngeal swabs and sputum specimens collected from hospitalized adults in rural Western Kenya
Source: Sci Rep. 2019 Aug 2;9:11237. doi: 10.1038/s41598-019-47713-4 (PMC6677726; doi:10.1038/s41598-019-47713-4)
Supplement: Supplementary file 1 — Supplementary Tables [file 41598_2019_47713_MOESM1_ESM.pdf]

Comparison of respiratory pathogen yields from Nasopharyngeal/Oropharyngeal swabs and sputum specimens collected from hospitalized adults in rural Western Kenya

Bryan O. Nyawanda[1*], Henry N. Njuguna[2], Clayton O. Onyango[3], Caroline Makokha[1], Shirley Lidechi[1], Barry Fields[2], Jonas M. Winchell[2], Jim S. Katieno[1], Jeremiah Nyaundi[1], Fredrick Ade[1], Gideon Emukule [4], Joshua A. Mott [2], Nancy Otieno[1], Marc-Alain Widdowson[3], Sandra S. Chaves[2,4]

[1] Kenya Medical Research Institute - Center for Global Health Research; [2] Centers for Disease Control and Prevention, Atlanta, GA, USA; [3] Division of Global Health Protection, Centers for Disease Control and Prevention, Kenya; [4] Influenza Program, Centers for Disease Control and Prevention, Kenya

***Corresponding author:**
Email: oteebryan@gmail.com or bnyawanda@kemricdc.org
Phone: +254 -725-765138
Kenya Medical Research Institute - P.O BOX 1578 – 40100 Kisumu – Kenya
https://orcid.org/0000-0003-0069-1473

Supplementary Table S1: Comparison of respiratory pathogen yields in nasopharyngeal/ oropharyngeal vs sputum specimens among HIV+ and HIV- patients, March 2014 – July 2015

Pathogens	HIV Positive n=115		p-value	HIV Negative n=179		p-value
	NPOP n (%)	Sputum n (%)		NPOP n (%)	Sputum n (%)	
Any pathogen	84 (73)	70 (61)	0.03	116 (65)	100 (56)	0.07
Any Bacterial pathogen	64 (56)	55 (48)	0.14	77 (43)	86 (48)	0.30
Any Viral pathogen	45 (39)	19 (17)	<0.01	70 (39)	26 (15)	<0.01
**Bacterial pathogens**						
Streptococcus pneumoniae	36 (31)	27 (23)	0.05	40 (22)	34 (19)	0.29
Streptococcus pyogenes	0	1 (1)		1 (1)	1 (1)	
Haemophilus influenzae- All	25 (22)	16 (14)	0.04	30 (17)	31 (17)	1.00
Haemophilus influenzae B 1	3 (3)	1 (1)	0.50	1 (1)	2 (1)	1.00
Haemophilus influenzae B 2	1 (1)	2 (2)	1.00	2 (1)	2 (1)	
Staphylococcus aureus	21 (18)	12 (10)	0.06	20 (11)	16 (9)	0.56
Pseudomonas aeroginosa	11 (10)	6 (5)	0.30	7 (4)	13 (7)	0.21
Legionella spp.	0	0		0	0	
Klebsiella pneumoniae	5 (4)	11 (10)	0.15	8 (4)	16 (9)	0.08
Bordetella pertussis	0	0		0	0	
Mycoplasma pneumoniae	1 (1)	0		0	0	
Chlamydia pneumoniae	0	0		1 (1)	0	
Moraxella catarrhalis	16 (14)	10 (9)	0.07	19 (11)	12 (7)	0.12
**Viral pathogens**						
Influenza A	1 (1)	2 (2)	0.99	11 (6)	6 (3)	0.06
Influenza B	2 (2)	2 (2)	1.00	1 (1)	2 (1)	0.99
Para-influenza 1	0	0		1 (1)	0	
Para-influenza 2	4 (3)	1 (1)	0.25	1 (1)	0	
Para-influenza 3	1 (1)	0		4 (2)	2 (1)	0.50
Para-influenza 4	0	0		2 (1)	0	
Resp. syncytial virus (RSV)	3 (3)	0		8 (4)	2 (1)	0.07
Rhinovirus	26 (23)	11 (10)	<0.01	37 (21)	12 (7)	<0.01
Enterovirus	4 (3)	0		6 (3)	0	
Human metapneumovirus	0	0		5 (3)	2 (1)	0.25
Adenovirus	1 (1)	2 (2)	1.00	0	2 (1)	
HCoV 1 (229E)	0	0		0	0	
HCoV 2 (NL63)	9 (8)	1 (1)	0.01	0	0	
HCoV 3 (OC43)	3 (3)	1 (1)	0.50	3 (2)	0	
HCoV 4 (HKU1)	0	0		3 (2)	0	
**Mycobacteria**						
Mycobacterium tuberculosis	1 (1)	12 (10)	<0.01	2 (1)	4 (2)	0.63
**Fungi**						
Pneumocystis jirovecii	0	0		0	0	

Supplementary Table S2: Comparison of respiratory pathogen yields in nasopharyngeal/ oropharyngeal vs sputum specimens among patients with and without malaria, March 2014 – July 2015

Pathogens	Malaria Positive n=57		p-value	Malaria Negative n=237		p-value
	NPOP n (%)	Sputum n (%)		NPOP n (%)	Sputum n (%)	
Any pathogen	38 (67)	27 (47)	0.03	162 (68)	143 (60)	0.04
Any Bacterial pathogen	23 (40)	22 (39)	1.00	118 (50)	119 (50)	1.00
Any Viral pathogen	23 (40)	10 (18)	<0.01	92 (39)	35 (15)	<0.01
**Bacterial pathogens**						
Streptococcus pneumoniae	12 (21)	6 (11)	0.03	64 (27)	55 (23)	0.16
Streptococcus pyogenes	0	1 (2)		1 (0)	1 (0)	
Haemophilus influenzae- All	7 (12)	6 (11)	1.00	48 (20)	41 (17)	0.30
Haemophilus influenzae B 1	0	0		4 (2)	3 (1)	1.00
Haemophilus influenzae B 2	0	0		3 (1)	4 (2)	1.00
Staphylococcus aureus	7 (12)	3 (5)	0.29	34 (14)	25 (11)	0.19
Pseudomonas aeroginosa	1 (2)	7 (12)	0.03	17 (7)	12 (5)	0.42
Legionella spp.	0	0		0	0	
Klebsiella pneumoniae	2 (4)	4 (7)	0.69	11 (5)	23 (10)	0.02
Bordetella pertussis	0	0		0	0	
Mycoplasma pneumoniae	0	0		1 (0)	0	
Chlamydia pneumoniae	0	0		1 (0)	0	
Moraxella catarrhalis	4 (7)	1 (2)	0.25	31 (13)	21 (9)	0.04
**Viral pathogens**						
Influenza A	3 (5)	2 (4)	1.00	9 (4)	5 (2)	0.22
Influenza B	0	0		3 (1)	4 (2)	1.00
Para-influenza 1	1 (2)	0		0	0	
Para-influenza 2	0	0		5 (2)	1 (0)	0.13
Para-influenza 3	0	0		5 (2)	2 (1)	0.25
Para-influenza 4	0	0		2 (1)	0	
Resp. syncytial virus (RSV)	2 (4)	1 (2)	1.00	9 (4)	1 (0)	0.02
Rhinovirus	14 (25)	5 (9)	<0.01	49 (21)	18 (8)	<0.01
Enterovirus	3 (5)	0		7 (3)	0	
Human metapneumovirus	0	0		5 (2)	2 (1)	0.25
Adenovirus	0	1 (2)		1 (0)	3 (1)	0.63
HCoV 1 (229E)	0	0		0	0	
HCoV 2 (NL63)	1 (2)	0		8 (3)	1 (0)	0.02
HCoV 3 (OC43)	2 (4)	1 (2)	1.00	4 (2)	0	
HCoV 4 (HKU1)	0	0		3 (1)	0	
**Mycobacteria**						
Mycobacterium tuberculosis	0	1 (2)		3 (1)	15 (6)	<0.01
**Fungi**						
Pneumocystis jirovecii	0	0		0	0	

Supplementary Table S3. Comparison of respiratory pathogen yields in nasopharyngeal/ oropharyngeal vs sputum specimens by disease severity*, March 2014 – July 2015

**Pathogens**	**Severe n=88**		**p-value**	**Non-severe n=206**		**p-value**
	**NPOP n (%)**	**Sputum n (%)**		**NPOP n (%)**	**Sputum n (%)**	
Any pathogen	55 (63)	60 (68)	0.44	145 (70)	110 (53)	<0.01
Any Bacterial pathogen	43 (49)	48 (55)	0.47	98 (48)	93 (45)	0.60
Any Viral pathogen	24 (27)	13 (15)	0.01	91 (44)	32 (16)	<0.01
**Bacterial pathogens**						
Streptococcus pneumoniae	24 (27)	20 (23)	0.34	52 (25)	41 (20)	0.06
Streptococcus pyogenes	0	1 (1)		1 (1)	1 (1)	1.00
Haemophilus influenzae- All	17 (19)	13 (15)	0.39	38 (18)	34 (17)	0.57
Haemophilus influenzae B 1	2 (2)	2 (2)		2 (1)	1 (0)	1.00
Haemophilus influenzae B 2	2 (2)	3 (3)	1.00	1 (0)	1 (0)	
Staphylococcus aureus	12 (14)	10 (11)	0.80	29 (14)	18 (9)	0.06
Pseudomonas aeroginosa	5 (6)	6 (7)	1.00	13 (6)	13 (6)	1.00
Legionella spp.	0	0		0	0	
Klebsiella pneumoniae	8 (9)	13 (15)	0.30	5 (2)	14 (7)	0.02
Bordetella pertussis	0	0		0	0	
Mycoplasma pneumoniae	0	0		1 (1)	0	
Chlamydia pneumoniae	1 (1)	0		0	0	
Moraxella catarrhalis	12 (14)	10 (11)	0.69	23 (11)	12 (6)	0.01
**Viral pathogens**						
Influenza A	3 (3)	1 (1)	0.50	9 (4)	6 (3)	0.38
Influenza B	1 (1)	1 (1)		2 (1)	3 (1)	1.00
Para-influenza 1	0	0		1 (1)	0	
Para-influenza 2	3 (3)	0		2 (1)	1 (1)	1.00
Para-influenza 3	1 (1)	1 (1)		4 (2)	1 (1)	0.25
Para-influenza 4	0	0		2 (1)	0	
Resp. syncytial virus (RSV)	3 (3)	1 (1)	0.50	8 (4)	1 (1)	0.04
Rhinovirus	10 (11)	7 (8)	0.45	53 (26)	16 (8)	<0.01
Enterovirus	2 (2)	0		8 (4)	0	
Human metapneumovirus	0	0		5 (2)	2 (1)	0.25
Adenovirus	1 (1)	2 (2)	1.00	0	2 (1)	
HCoV 1 (229E)	0	0		0	0	
HCoV 2 (NL63)	3 (3)	1 (1)	0.50	6 (3)	0	
HCoV 3 (OC43)	0	0		6 (3)	1 (1)	0.06
HCoV 4 (HKU1)	1 (1)	0		2 (1)	0	
**Mycobacteria**						
Mycobacterium tuberculosis	1(1)	10 (11)	0.01	2 (1)	6 (3)	0.13
**Fungi**						
Pneumocystis jirovecii	0	0		0	0	

*Disease severity assessed by prolonged hospitalization (severe = >7 days in hospital; non-severe = ≤7 days in hospital).

Supplementary Table S4. Comparison of respiratory pathogen yields in nasopharyngeal/ oropharyngeal vs sputum specimens by duration of illness*, March 2014 – July 2015

**Pathogens**	**Acute illness n=116**		**p-value**	**Non-acute illness n=178**		**p-value**
	**NPOP n (%)**	**Sputum n (%)**		**NPOP n (%)**	**Sputum n (%)**	
Any pathogen	80 (69)	65 (56)	0.02	120 (67)	105 (59)	0.08
Any Bacterial pathogen	53 (46)	53 (46)	1.00	88 (49)	88 (49)	1.00
Any Viral pathogen	48 (41)	21 (18)	<0.01	67 (38)	24 (13)	<0.01
**Bacterial pathogens**						
Streptococcus pneumoniae	28 (24)	25 (22)	0.61	48 (27)	36 (20)	0.02
Streptococcus pyogenes	1 (1)	1 (1)		0	1 (1)	
Haemophilus influenzae- All	20 (17)	20 (17)	1.00	35 (20)	27 (15)	0.13
Haemophilus influenzae B 1	0	1 (1)		4 (2)	2 (1)	0.50
Haemophilus influenzae B 2	0	1(1)		3 (2)	3 (2)	
Staphylococcus aureus	17 (15)	9 (8)	0.12	24 (13)	19 (11)	0.42
Pseudomonas aeroginosa	6 (5)	8 (7)	0.75	12 (7)	11 (6)	1.00
Legionella spp.	0	0		0	0	
Klebsiella pneumoniae	7 (6)	8 (7)	1.00	6 (3)	19 (11)	0.01
Bordetella pertussis	0	0		0	0	
Mycoplasma pneumoniae	1 (1)	0		0	0	
Chlamydia pneumoniae	1 (1)	0		0	0	
Moraxella catarrhalis	13 (11)	9 (8)	0.29	22 (12)	13 (7)	0.04
**Viral pathogens**						
Influenza A	6 (5)	4 (3)	0.50	6 (3)	3 (2)	0.38
Influenza B	0	0		3 (2)	4 (2)	1.00
Para-influenza 1	1 (1)	0		0	0	
Para-influenza 2	1 (1)	1 (1)		4 (2)	0	
Para-influenza 3	2 (2)	1 (1)	1.00	3 (2)	1 (1)	0.50
Para-influenza 4	1 (1)	0		1 (1)	0	
Resp. syncytial virus (RSV)	2 (2)	0		9 (5)	2 (1)	0.04
Rhinovirus	32 (28)	12 (10)	<0.01	31 (17)	11 (6)	<0.01
Enterovirus	4 (3)	0		6 (3)	0	
Human metapneumovirus	3 (3)	2 (2)	1.00	2 (1)	0	
Adenovirus	0	2 (2)		1 (1)	2 (1)	1.00
HCoV 1 (229E)	0	0		0	0	
HCoV 2 (NL63)	1 (1)	0		8 (4)	1 (1)	0.02
HCoV 3 (OC43)	1 (1)	0		5 (3)	1 (1)	0.13
HCoV 4 (HKU1)	1 (1)	0		2 (1)	0	
**Mycobacteria**						
Mycobacterium tuberclosis	2 (2)	4 (3)	0.63	1 (1)	12 (7)	<0.01
**Fungi**						
Pneumocystis jirovecii	0	0		0	0	

*Duration of illness measured by time from illness onset to hospital admission (acute illness= <5 days; non-acute illness= ≥5 days)

Supplementary Table S5. Co-detection of respiratory pathogens from nasopharyngeal/ oropharyngeal swabs

Pathogens	Code	Bacterial pathogens													Viral pathogens																mytb	pnji
		stpn	gast	hiat	hitb1	hitb2	stau	psae	lspp	klpn	bop1	mypn	chpn	moca	FluA	FluB	piv1	piv2	piv3	piv4	resv	rhiv	entv	hmpv	aden	hcv1	hcv2	hcv3	hcv4		
**Bacterial pathogens**																																
Streptococcus pneumoniae	stpn	76																														
Streptococcus pyogenes	gast	1	1																													
Haemophilus influenzae- All	hiat	29	1	55																												
Haemophilus influenzae B 1	hitb1	2	0	3	4																											
Haemophilus influenzae B 2	hitb2	0	0	2	1	3																										
Staphylococcus aureus	stau	12	0	10	0	0	41																									
Pseudomonas aeroginosa	psae	8	0	4	0	0	4	18																								
Legionella spp.	lspp	0	0	0	0	0	0	0	0																							
Klebsiella pneumoniae	klpn	6	0	4	0	0	3	1	0	13																						
Bordetella pertussis	bop1	0	0	0	0	0	0	0	0	0	0																					
Mycoplasma pneumoniae	mypn	0	0	0	0	0	1	1	0	0	0	1																				
Chlamydia pneumoniae	chpn	0	0	0	0	0	1	0	0	0	0	0	1																			
Moraxella catarrhalis	moca	19	0	18	2	0	5	3	0	3	0	0	1	35																		
**Viral pathogens**																																
Influenza A	FluA	4	0	3	0	0	1	0	0	0	0	0	0	2	12																	
Influenza B	FluB	0	0	0	0	0	0	0	0	0	0	0	0	0	0	3																
Para-influenza 1	piv1	0	0	1	0	0	0	0	0	1	0	0	0	0	0	0	1															
Para-influenza 2	piv2	2	0	1	0	0	3	0	0	0	0	0	0	1	0	0	0	5														
Para-influenza 3	piv3	2	0	3	0	1	0	1	0	0	0	0	0	0	0	0	0	0	5													
Para-influenza 4	piv4	1	0	2	0	0	0	0	0	0	0	0	0	0	1	0	0	0	0	2												
Resp. syncytial virus (RSV)	resv	4	0	2	0	0	2	1	0	0	0	0	0	3	2	0	0	0	0	0	11											
Rhinovirus	rhiv	19	0	19	1	1	11	2	0	2	0	0	0	7	0	0	0	0	2	1	0	63										
Enterovirus	entv	2	0	1	0	0	0	1	0	0	0	1	0	0	0	0	0	0	1	0	0	6	10									
Human metapneumovirus	hmpv	0	0	2	0	0	1	0	0	1	0	0	0	2	0	0	0	0	0	0	0	2	0	5								
Adenovirus	aden	0	0	0	0	0	0	0	0	0	0	0	0	0	0	0	0	0	0	0	0	0	0	0	1							

HCoV 1 (229E)	hcv1	0	0	0	0	0	0	0	0	0	0	0	0	0	0	0	0	0	0	0	0	0	0	0	0	0					
HCoV 2 (NL63)	hcv2	3	0	3	0	0	0	1	0	0	0	0	0	3	0	0	0	0	0	0	0	4	1	0	0	0	9				
HCoV 3 (OC43)	hcv3	2	0	1	0	0	1	1	0	0	0	0	0	0	0	0	0	0	0	0	0	1	0	1	0	0	0	6			
HCoV 4 (HKU1)	hcv4	1	0	1	0	0	0	0	0	0	0	0	0	0	1	0	0	0	0	0	1	0	0	0	0	0	0	0	3		
**Mycobacteria**																															
Mycobacterium tuberclosis	mytb	0	0	1	0	0	1	0	0	1	0	0	0	0	0	0	0	0	0	0	0	1	1	0	0	0	0	0	0	3	
**Fungi**																															
Pneumocystis jirovecii	pnji	0	0	0	0	0	0	0	0	0	0	0	0	0	0	0	0	0	0	0	0	0	0	0	0	0	0	0	0	0	0

Supplementary Table S6. Co-detection of respiratory pathogens from sputum specimens

Pathogens	Code	Bacterial pathogens													Viral pathogens															mytb	pnji
		stpn	gast	hiat	hitb1	hitb2	stau	psae	lspp	klpn	bop1	mypn	chpn	moca	FluA	FluB	piv1	piv2	piv3	piv4	resv	rhiv	entv	hmpv	aden	hcv1	hcv2	hcv3	hcv4		
**Bacterial pathogens**																															
Streptococcus pneumoniae	stpn	61																													
Streptococcus pyogenes	gast	1	2																												
Haemophilus influenzae- All	hiat	23	1	47																											
Haemophilus influenzae B 1	hitb1	2	0	2	3																										
Haemophilus influenzae B 2	hitb2	2	0	3	1	4																									
Staphylococcus aureus	stau	6	1	4	0	0	28																								
Pseudomonas aeroginosa	psae	3	0	2	0	0	2	19																							
Legionella spp.	lspp	0	0	0	0	0	0	0	0																						
Klebsiella pneumoniae	klpn	6	0	6	0	0	5	2	0	27																					
Bordetella pertussis	bop1	0	0	0	0	0	0	0	0	0	0																				
Mycoplasma pneumoniae	mypn	0	0	0	0	0	0	0	0	0	0	0																			
Chlamydia pneumoniae	chpn	0	0	0	0	0	0	0	0	0	0	0	0																		
Moraxella catarrhalis	moca	14	0	11	1	2	2	2	0	2	0	0	0	22																	
**Viral pathogens**																															
Influenza A	FluA	1	0	2	0	0	0	1	0	0	0	0	0	0	7																
Influenza B	FluB	0	0	0	0	0	0	0	0	0	0	0	0	0	0	4															
Para-influenza 1	piv1	0	0	0	0	0	0	0	0	0	0	0	0	0	0	0	0														
Para-influenza 2	piv2	1	0	0	0	0	0	0	0	0	0	0	0	0	0	0	0	1													
Para-influenza 3	piv3	0	0	1	0	0	0	0	0	0	0	0	0	0	0	0	0	0	2												
Para-influenza 4	piv4	0	0	0	0	0	0	0	0	0	0	0	0	0	0	0	0	0	0	0											
Resp. syncytial virus (RSV)	resv	1	0	0	0	0	0	1	0	1	0	0	0	0	0	0	0	0	0	0	2										
Rhinovirus	rhiv	6	1	7	1	1	3	1	0	2	0	0	0	2	0	0	0	0	0	0	0	23									
Enterovirus	entv	0	0	0	0	0	0	0	0	0	0	0	0	0	0	0	0	0	0	0	0	0	0								
Human metapneumovirus	hmpv	0	0	2	0	0	0	0	0	1	0	0	0	0	0	0	0	0	0	0	0	1	0	2							
Adenovirus	aden	2	0	1	1	1	0	0	0	0	0	0	0	1	0	0	0	0	0	0	0	1	0	0	4						

HCoV 1 (229E)	hcv1	0	0	0	0	0	0	0	0	0	0	0	0	0	0	0	0	0	0	0	0	0	0	0	0	0					
HCoV 2 (NL63)	hcv2	0	0	0	0	0	0	0	0	1	0	0	0	0	0	0	0	0	0	0	0	0	0	0	0	0	1				
HCoV 3 (OC43)	hcv3	0	0	0	0	0	0	1	0	0	0	0	0	0	0	0	0	0	0	0	0	0	0	0	0	0	0	1			
HCoV 4 (HKU1)	hcv4	0	0	0	0	0	0	0	0	0	0	0	0	0	0	0	0	0	0	0	0	0	0	0	0	0	0	0	0		
**Mycobacteria**																															
Mycobacterium tuberclosis	mytb	0	0	0	0	0	2	0	0	5	0	0	0	0	0	0	0	0	0	0	0	0	0	0	0	0	1	0	0	16	
**Fungi**																															
Pneumocystis jirovecii	pnji	0	0	0	0	0	0	0	0	0	0	0	0	0	0	0	0	0	0	0	0	0	0	0	0	0	0	0	0	0	0
